# Molecular-Scale Visualization of Steric Effects of Ligand Binding to Reconstructed Au(111) Surfaces

**DOI:** 10.1021/jacs.4c00002

**Published:** 2024-04-16

**Authors:** Liya Bi, Sasawat Jamnuch, Amanda Chen, Alexandria Do, Krista P. Balto, Zhe Wang, Qingyi Zhu, Yufei Wang, Yanning Zhang, Andrea R. Tao, Tod A. Pascal, Joshua S. Figueroa, Shaowei Li

**Affiliations:** † Department of Chemistry and Biochemistry, University of California, San Diego, California 92093-0309, United States; ‡ Program in Materials Science and Engineering, University of California, San Diego, California 92093-0418, United States; § Department of Nano and Chemical Engineering, University of California, San Diego, California 92093-0448, United States; ∥ Institute of Fundamental and Frontier Sciences, University of Electronic Science and Technology of China, Chengdu 611731, China

## Abstract

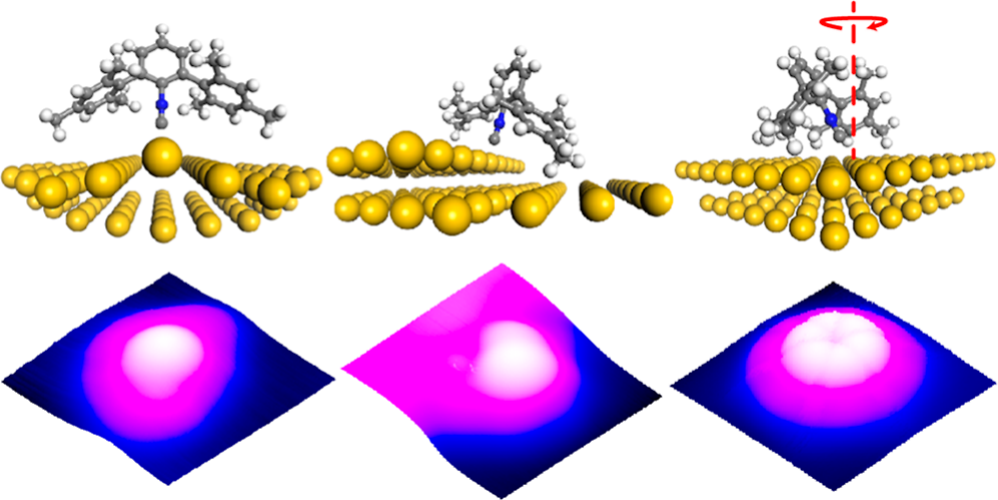

Direct imaging of single molecules at nanostructured interfaces is a grand challenge with potential to enable new, precise material architectures and technologies. Of particular interest are the structural morphology and spectroscopic signatures of the adsorbed molecule, where modern probes are only now being developed with the necessary spatial and energetic resolution to provide detailed information at the molecule–surface interface. Here, we directly characterize the adsorption of individual *m*-terphenyl isocyanide ligands on a reconstructed Au(111) surface through scanning tunneling microscopy and inelastic electron tunneling spectroscopy. The site-dependent steric pressure of the various surface features alters the vibrational fingerprints of the *m*-terphenyl isocyanides, which are characterized with single-molecule precision through joint experimental and theoretical approaches. This study provides molecular-level insights into the steric-pressure-enabled surface binding selectivity as well as its effect on the chemical properties of individual surface-binding ligands.

## Introduction

Steric interference refers to the repulsion between atoms or functional groups that are forced together by geometric limitations within or between molecules.^1−4^ Its origin is in the fundamental Pauli force, as well as long-range interactions between the dipole moments of adjacent atoms or functional groups,^5−8^ which creates a destabilizing force that can distort a molecule from its preferred geometry,^9,10^ induce dissociation or bond-breaking events,^11^ or inhibit chemical reactivity.^12−14^ Accordingly, the concept of steric interference has long-standing importance in the design, study, and understanding of chemical^1−4^ and biological^15^ systems. Indeed, the exploitation of steric interference has been central to the isolation of inherently reactive molecules^4,16−18^ and has underpinned the design of catalysts for asymmetric synthesis.^19^ Maximizing or minimizing steric interferences is also critical design principles for selective drug delivery to enzyme active sites.^20^ In addition, steric interference, whether in an intramolecular or intermolecular context, can significantly affect the conformational preferences of a molecule^21^ or the interactions between molecules,^22^ which in turn can impact physical properties such as melting point, boiling point, and solubility.

From a synthetic standpoint, the modulation of steric properties can provide an accessible control knob to achieve chemical selectivity. A molecule with designed steric features can preferably interact with other substrates in areas where steric repulsion is minimized.^23^ The ability to control reaction sites or regions can also enable the selective and predictable adsorption of molecules onto structured surfaces. This concept can be particularly useful in the field of nanotechnology, where the properties of nanostructures can be tuned by modifying their surface chemistry through the use of ligands.^24−29^ For example, the selective adsorption of ligands onto different facets of a nanocrystal can control its shape and size and, as a result, directly influence its optical, electronic, and catalytic properties.^30−33^ Furthermore, the potential to harness steric effects in the design of ligands that specifically target certain surface sites on a nanostructure would have a profound impact on the creation of highly selective and efficient catalysts for a range of chemical reactions.^34−37^ Therefore, utilizing and ultimately controlling steric effects is crucial for manipulating properties of nanostructures and developing new materials with tailored functionalities.

**Figure 1:**
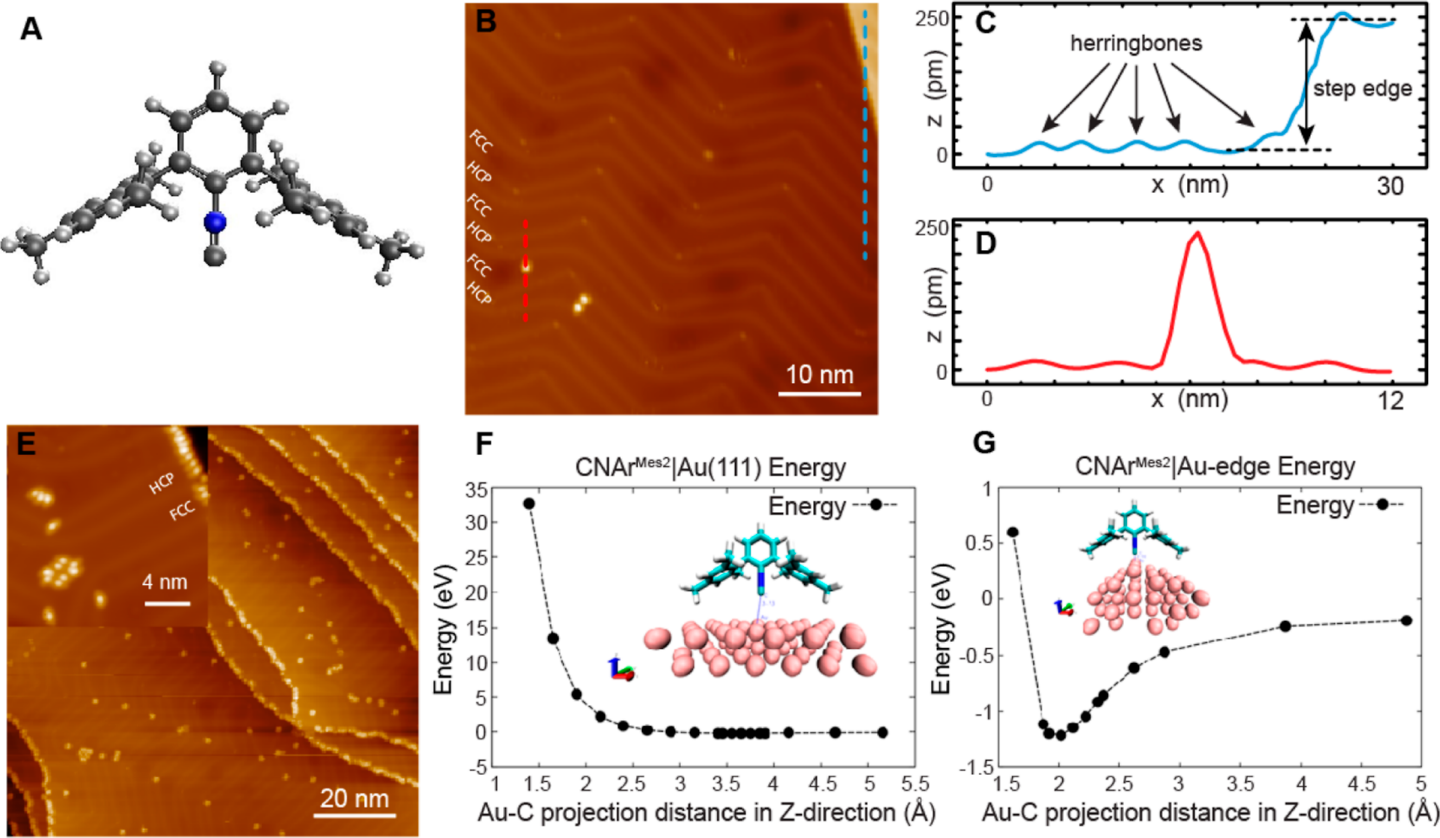
Thermally induced diffusion of the CNAr^Mes2^ ligand on Au(111). (A) Ball-and-stick model of CNAr^Mes2^, with N, C, and H atoms shown in blue, dark gray, and light gray, respectively. (B) Topographic image of randomly adsorbed CNAr^Mes2^ (an isolated one and a dimer) on Au(111) at 5 K. The FCC and HCP domains are labeled for clarity. (C) Line profile across the blue dashed line in (B), showing the diverse landscapes on Au(111). (D) Line profile across the isolated CNAr^Mes2^ in (B). (E) Topographic image of the surface with high-density CNAr^Mes2^ after warming up to room temperature and recooling to 5 K, with zoom-in inset showing CNAr^Mes2^ in clusters, on herringbone elbow sites, and at the step edge. (F,G) Simulated adsorption energy landscapes of CNAr^Mes2^ on Au(111) at the planar (F) and curved surface (G). Imaging parameters were set to −1 V, 100 pA for (B), 1 V, 100 pA for (E), and −500 mV, 50 pA for the inset of (E).

When a system is designed that is sensitive to ligand–surface steric interactions, it is important that the surface-binding group possesses sites of potential steric interference that are well-defined. *m*-Terphenyl isocyanides (Figure 1A) are a promising class of ligands in this regard due to their strong surface binding ability and their unique and modifiable steric profile.^38−41^ These ligands comprise an aryl isocyanide (i.e., CNAr; Ar = aryl) binding group, which has been long established to bind to metal surfaces.^42−47^ In the *m*-terphenyl modification, the isocyanide group is flanked by two additional, mutually meta, encumbering arenes, which create significant steric interference and pressure in the direction pointing toward the metal surface. This steric pressure is maximized when the *m*-terphenyl isocyanide ligand is bound to a planar metal surface. However, it can be significantly reduced when the ligand is bound to a convex surface, such as the step edge on a metal surface, where the *m*-terphenyl group can localize in a less sterically hindered binding environment. Recent spectroscopic studies have provided evidence of preferable adsorption of *m*-terphenyl isocyanide ligands to nanocrystal surfaces exhibiting high degrees of nanocurvature.^23^ Specifically, it was shown that the *m*-terphenyl isocyanide (Figure 1A), CNAr^Mes2^ (Ar^Mes2^ = 2,6-(2,4,6-Me_3_C_6_H_2_)_2_C_6_H_3_), could readily bind to Au nanospheres (AuNSs) with diameters between 5 and 50 nm but did not bind to larger diameter particles with lower degrees of nanocurvature to an appreciable extent. This sterically induced binding selectivity enabled the development of a chemical method for nanoparticle separation based on size as well as a chemical means of affecting nanoparticle size-focusing. However, while this ensemble-level study elucidated the global effects of CNAr^Mes2^ binding to AuNS surfaces, direct visualization of this binding as well as information concerning the precise steric interactions between the ligands and their nanoscale environment were absent. Here, we fill in this gap with molecular-scale characterization of steric-pressure-induced site-selective binding of individual *m*-terphenyl isocyanide ligands to a reconstructed Au(111) surface using scanning tunneling microscopy (STM),^48,49^ inelastic electron tunneling spectroscopy (IETS),^50,51^ and computational simulations. The results presented here provide a detailed structural and spectroscopic picture of the role of steric effects at the ligand–surface interface.

## Results and Discussion

### Visualization of Selective Binding of CNAr^Mes2^ to Au(111)

Here, we study the binding of CNAr^Mes2^ to the reconstructed Au(111) surfaces. We choose Au(111) as the substrate because of its diverse surface structures, including high-curvature step edges as well as alternating face center cubic (FCC) and hexagonal close-packed (HCP) facets separated by protruding herringbone reconstructions.^52,53^ As shown in Figure 1B,C, each topological site possesses inherently distinct nanocurvature that can be resolved by STM topographic imaging (3D view in Figure S1B). In addition, the distinct curvature of these sites leads to an inhomogeneous steric environment upon molecular adsorption. Importantly, both the step edges and herringbone sites have convex curvature, which is expected to exhibit low degrees of steric pressure on bound ligands.

To understand the baseline profile of CNAr^Mes2^ adsorption, atomically clean Au(111) surfaces were dosed with the evaporated ligand at 5 K and 10^–10^ Torr in situ at the STM junction. Initial STM topographical images taken at this temperature (Figure 1B) revealed that the CNAr^Mes2^ ligands are adsorbed randomly on the Au(111) surface without any discernible preference for a particular site. Even at very low-molecular coverage, isolated CNAr^Mes2^ molecules can be seen on both the FCC and HCP basal plane between herringbones, protruding at a height of about 250 pm (Figure 1D). At such a low temperature, it is expected that the CNAr^Mes2^ molecules rapidly release thermal energy to the environment and remain close to the location of the initial deposition. To probe for thermally induced migration events, especially to energetically favorable adsorption sites, the Au(111) substrate with either low or high CNAr^Mes2^ coverage was brought to room temperature and subsequently recooled to 5 K. Topographical images taken afterward indicated clear migration of the CNAr^Mes2^ molecules within the totality of the substrate and no individual CNAr^Mes2^ left on either FCC or HCP basal plane (Figure 1E for high and Figure S2C for low CNAr^Mes2^ coverage case). When the initial coverage is low, CNAr^Mes2^ shows an obvious preference to the step-edge positions in the FCC domain over any other surface sites after warming to room temperature (Figure S2C). When the initial density is high, while some CNAr^Mes2^ ligands can be found on the herringbone elbow sites after warming to room temperature, the vast majority of the molecules migrate to the step-edge positions in both the FCC and HCP domains (Figure 1E). Notably, previous studies have identified herringbone elbows as the most chemically reactive sites for ligand binding on Au(111) surfaces, followed by FCC domains.^54−56^ In contrast, the HCP facets and step edges both exhibit relative inertness. However, our findings clearly indicate a preference of CNAr^Mes2^ ligands for adsorbing on HCP step edges over herringbone elbows (as shown in the inset of Figure 1E). This observation strongly suggests the presence of an energetic factor beyond metal–ligand binding considerations, which is attributed to reduced steric repulsion resulting from the large convex surface curvature at the edge sites and is supported by theoretical calculations. Indeed, as shown in Figure 1F, the adsorption energy of the molecule on the basal plane is nearly negligible, while a ∼1.2 eV deep potential well exists at the Au edge (Figure 1G), and is characterized by a ∼2 Å long Au–C bond. This calculated Au–C bond length is consistent with those found for structurally characterized molecular gold–isocyanide complexes [average *d*(Au–C) = 1.964 ± 0.026 Å; Figure S3B].^57^ Moreover, we also observed molecular clusters on the FCC domains after warming the sample up to room temperature, indicating that the intermolecular interaction could also alter either the steric pressure or the ligand–metal interaction, which in turn affect the molecular binding behavior (Figures 1E inset and S4A–D).

### Impact of Steric Effects on the Structure of Adsorbed CNAr^Mes2^ on Au(111)

**Figure 2:**
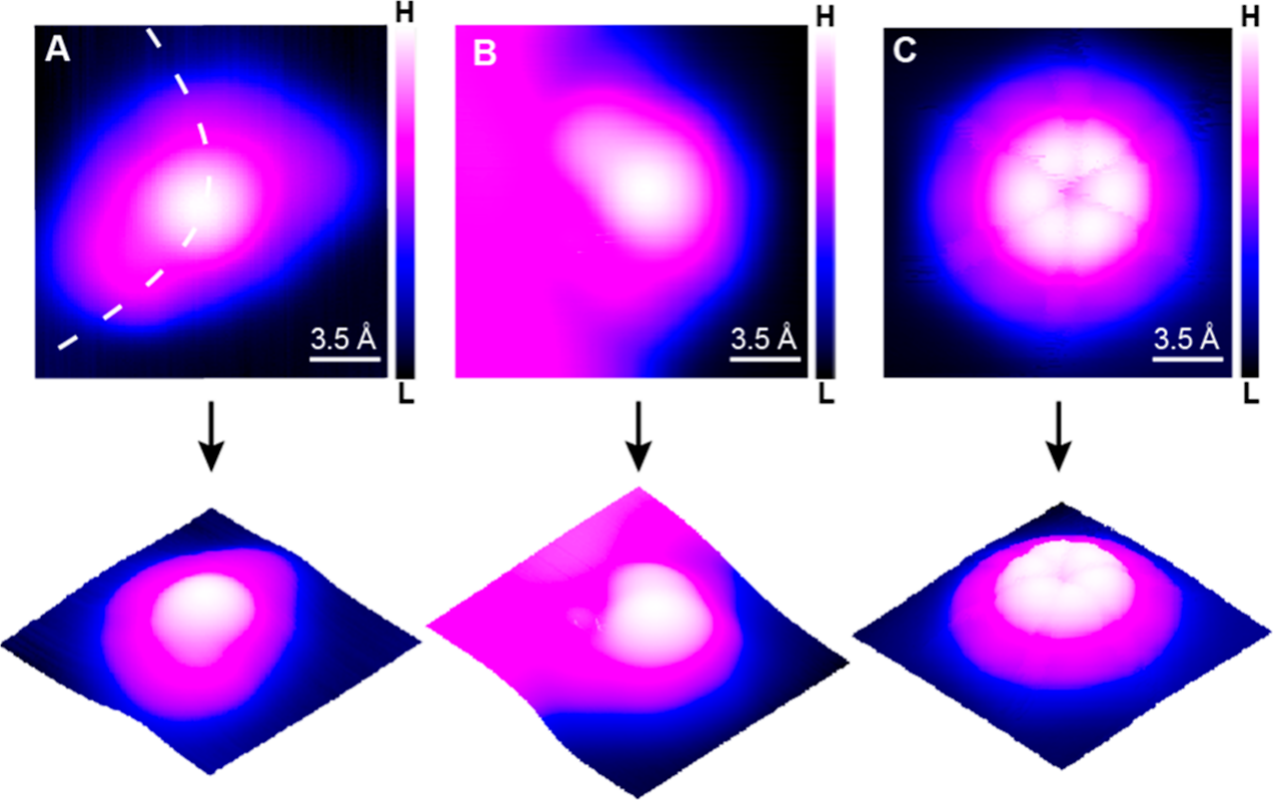
Visualizing the impact of steric effects on the adsorption structure of a single CNAr^Mes2^. (A–C) Topographic images of individual CNAr^Mes2^ herringbone elbow site (A), step edge (B), and planar surface (C). The white dashed curve in (A) indicates the herringbone. The bottom panel of each figure is the three dimensional visualization of different adsorption geometries of CNAr^Mes2^. Imaging parameters were set to (A) −50 mV, 20 pA and (B,C) −85 mV, 100 pA.

The STM images of individual CNAr^Mes2^ ligands adsorbed at different sites provide additional evidence of the influence of steric effects on the molecular adsorption structure and migration kinetics. Shown in Figure 2A–C are the topographic images of the isolated CNAr^Mes2^ ligands on the herringbone elbow site, at the step edge, and on the FCC basal plane, respectively (see also isolated CNAr^Mes2^ on the HCP basal plane in Figure S5). As shown in Figure 2A, the molecule is situated on top of the herringbone elbow site and appears crescent-shaped, less symmetric than its molecular structure (Figure 1A). This indicates that the molecule tilts to one side upon its adsorption on the herringbone elbow site (Figure S2D), in good agreement with the ∼10° tilt angle given by computational simulations (Figure S6A). At the step edge, the molecule appears to straddle the edge with one mesityl group on the upper Au layer and the other on the lower layer (Figures 2 and S2E), with a calculated tilting angle of ∼32° relative to the (111) direction (Figure S6B). We find that molecular adsorption at the step edge is highly stable, while adsorption on top of the herringbone elbow site can change conformations when disturbed by either the STM tip or tunneling electrons (Figure S4C–F). This observation agrees with the notion that the herringbone elbow sites, which possess an intermediate degree of surface curvature, also present greater ligand–surface steric pressures in comparison to the step edge sites. Such a steric bulk-induced bond weakening is most evident in the absence of any convex curvature. The images of isolated CNAr^Mes2^ ligands adsorbed on both FCC and HCP basal planes show a six-lobe feature (Figures 2C and S5), presenting a rapidly switching/rotating behavior among six equivalent adsorption geometries which are defined by the symmetry of Au(111). This is consistent with our molecular dynamics (MD) simulations at 5 K, which shows a CNAr^Mes2^ on the planar surface undergoing hindered rotations but only vibrating molecules on the herringbones and at the step edges (Movies S1, S2 and S3). Importantly, these simulations reveal that the steric pressure between the CNAr^Mes2^ ligands and the surface perturbs the binding to such an extent that, even at a low temperature of 5 K, thermal energy is sufficient to excite rotational motion around the metal-binding isocyanide group. This was further confirmed by calculating the in-plane and out-of-plane rotational temperatures from MD simulations (Table S1), where we find populated low-energy rotational states for the molecule on the planar surface at 5 K, which are not populated for molecules at the herringbone or step edges.

### Imaging Surface-Induced Vibrational Modes of CNAr^Mes2^ on Au(111)

**Figure 3:**
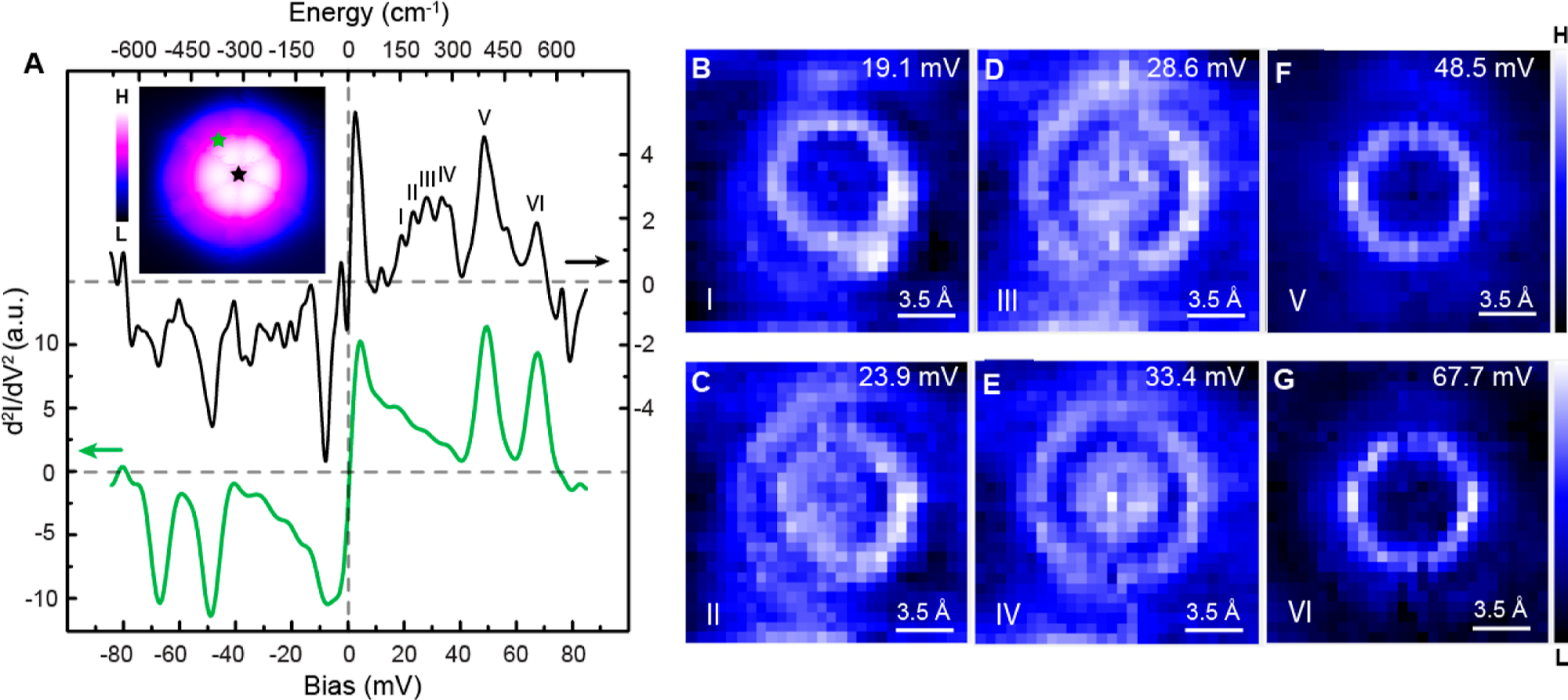
Vibrational characterization of individual CNAr^Mes2^. (A) IETS spectra of the molecule (inset) at two different positions, indicated by black and green stars, respectively. The spectra are vertically shifted for clarity. Imaging and spectroscopy parameters were −85 mV, 100 pA and −85 mV, 1.2 nA, respectively. (B–G) d^2^*I*/d*V*^2^ mapping of the same CNAr^Mes2^ at six different bias voltages, ranging from 19.1 to 67.7 mV. Imaging parameters for the mapping were set to −67.7 mV, 400 pA.

Remarkably, the spectroscopic properties of the individual CNAr^Mes2^ ligands are sensitive to the variation in steric pressures at its unique surface binding environment and are evident from modifications on their molecular vibrational fingerprints. To probe the vibrational features of CNAr^Mes2^ ligands at different binding sites, IETS^50,51^ was utilized, which is a highly efficient technique for investigating low-energy molecular vibrations at a submolecular scale. The low-energy vibrational modes are highly sensitive to the molecule–surface interaction and thus are a valuable means of characterizing the variations in molecular properties that occur in response to local chemical environments. IETS measures the second derivative of the tunneling current with respect to bias (d^2^*I*/d*V*^2^). A pair of symmetric peak and dip observed over the origin of the spectra indicates the bias corresponding to an inelastic excitation, such as molecular vibration,^50,58^ rotation,^59,60^ or spin excitation.^61,62^ The d^2^*I*/d*V*^2^ spectra obtained from an isolated CNAr^Mes2^ ligand on the FCC basal plane exhibits rich vibrational features below 85 meV/685.6 cm^–1^ with the intensity of the peaks and dips varying within the molecule (labeled with I–VI in Figure 3A). Since STM-IETS detects the conductance change of the junction due to the vibrational excitation, its spatial distribution closely resembles the nuclear motions. Figure 3A depicts that the signals emanating from the ∼49 and ∼67 meV vibrational modes (modes V and VI) are highly conspicuous and away from the center of the molecule (green spectrum). Conversely, the lower energy modes between ∼18 and ∼34 meV (modes I–IV) exhibit a more robust signal intensity near the center of the molecule (black spectrum). The spatially resolved mappings of the d^2^*I*/d*V*^2^ signal (Figures 3B–G and S7) provide an intuitive microscopic visualization of the vibrational motions. The images captured at 23.9 28.6, and 33.4 mV (modes II, III, and IV) exhibit a bullseye feature, which provides a distinctive motion pattern involving both the central aryl ring and the two *para*-methyl groups. In contrast, the images taken at 19.1, 48.5, and 67.7 mV (modes I, V, and VI) display a donut shape whose diameter, ∼7 Å, is close to the separation between carbon atoms in the *ortho*-methyl groups on different mesityl groups of a free-standing CNAr^Mes2^, indicating the primary motion of four *ortho*-methyl groups. It is worth mentioning that the d^2^*I*/d*V*^2^ images taken with biases below 30 mV do not closely follow the molecular symmetry. This phenomenon can be attributed to the coupling of vibrational excitation with the surface state of Au(111). Specifically, the Friedel oscillation resulting from the scattering of low-energy electrons near Au Fermi level leads to a spatial variation in the electron density of states,^63,64^ which in turn breaks the symmetry of the excitation cross section of the molecular vibration.

**Figure 4:**
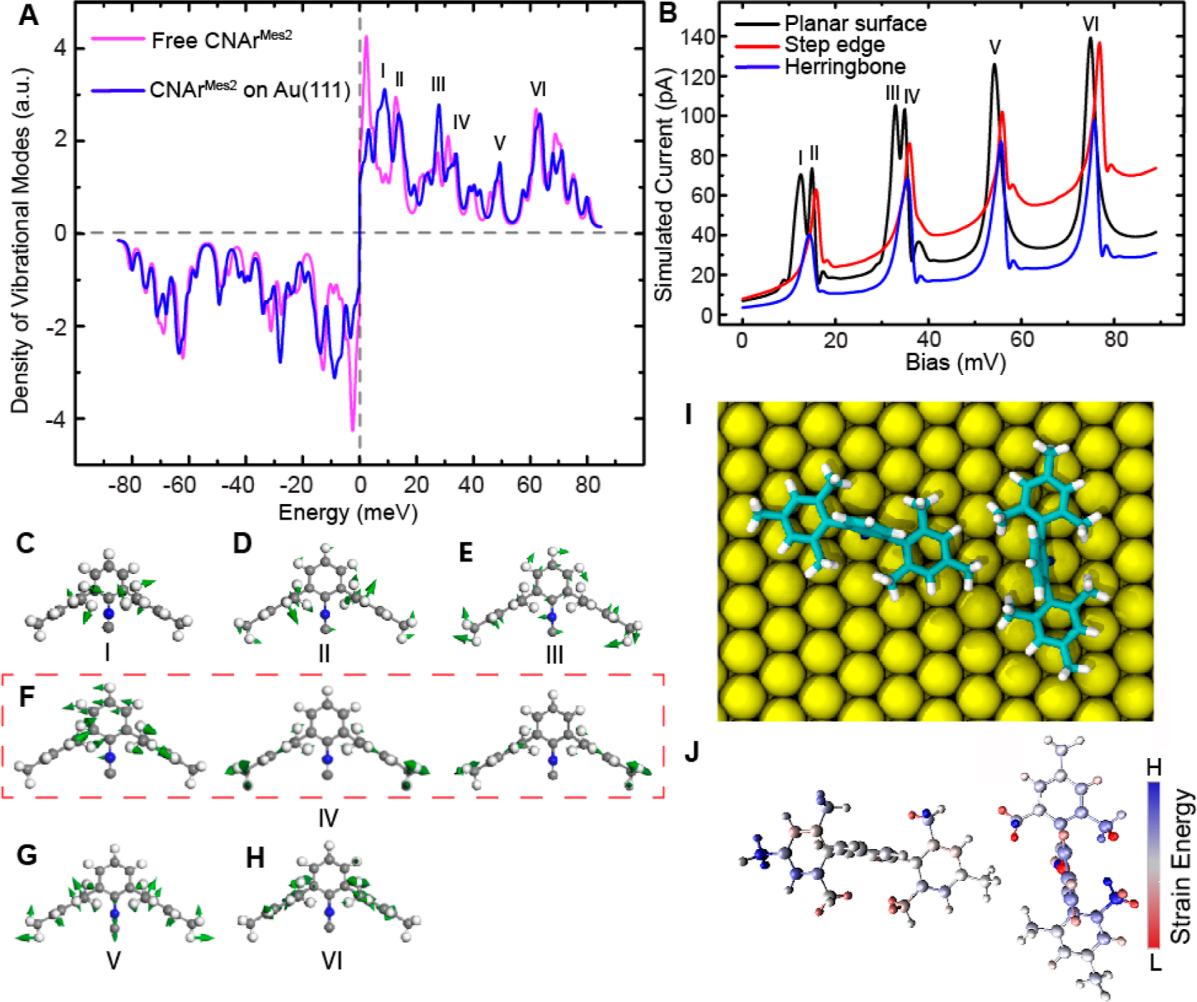
Theoretically calculated vibrational modes of individual CNAr^Mes2^ and strain distribution within a CNAr^Mes2^ dimer. (A) Calculated density of vibrational modes of free CNAr^Mes2^ (magenta) and CNAr^Mes2^ on Au(111) (blue). (B) Simulated elastic tunneling current change due to the vibrational excitation of CNAr^Mes2^ on the planar surface (black), at the step edge (red), and on herringbone (blue) of Au(111). (C–H) Simulated nuclear motions within free CNAr^Mes2^. (I) Top view of a simulated CNAr^Mes2^ dimer on Au(111). (J) Top view of the simulated strain energy distribution within the CNAr^Mes2^ dimer in (I). Blue (red) means increased (decreased) strain compared with the monomer.

By comparing the microscopic patterns with theoretical simulations (Figures 4A–H and S8), we can clearly identify the detailed molecular motion corresponding to the experimentally observed vibrational modes. Due to the structural complexity of the CNAr^Mes2^ ligand, rich features are exhibited in the simulated vibrational density (Figure 4A). Six vibrational modes stand out in the simulated elastic tunneling current (Figure 4B and Supporting Information) due to their relatively large out-of-plane nuclear motions, resulting in a stronger impact on junction conductance. It is important to note that the slight difference in energy for the simulated vibrational mode when compared between Figure 4A,B is due to the introduction of a top gold electrode in the simulation of the latter, as depicted in Figure S9. The energies and spatial distribution of these vibrational modes closely agree with the features measured experimentally with STM-IETS. Figure 4C–H presents the simulated nuclear motions within CNAr^Mes2^ in response to the molecular vibrational modes imaged in Figure 3B–G. The low-energy modes I–IV (Figure 4C–F) involve the frustrated rotational motion of different portions of CNAr^Mes2^. It is noteworthy that we attribute mode IV to the superposition of three vibrations (Figure 4F) of CNAr^Mes2^ due to their similar energies. The hindered rotation of the central aryl ring (Figure 4F, left) and the stretching motion of the para-methyl groups (Figure 4F, middle and right) together contribute to the bullseye feature in the d^2^*I*/d*V*^2^ mapping at 33.4 mV (Figure 3E). The vibration at 48.5 meV is the bouncing motion (Figure 4G) of CNAr^Mes2^ on the surface. The 67.7 meV mode pertains to the collective bending of C–H bonds within the four *ortho*-methyl groups (Figure 4H) and is therefore denoted as the *ortho*-methyl twisting mode.

### Impact of Steric Effects on the Chemical Properties of CNAr^Mes2^ on Au(111)

**Figure 5:**
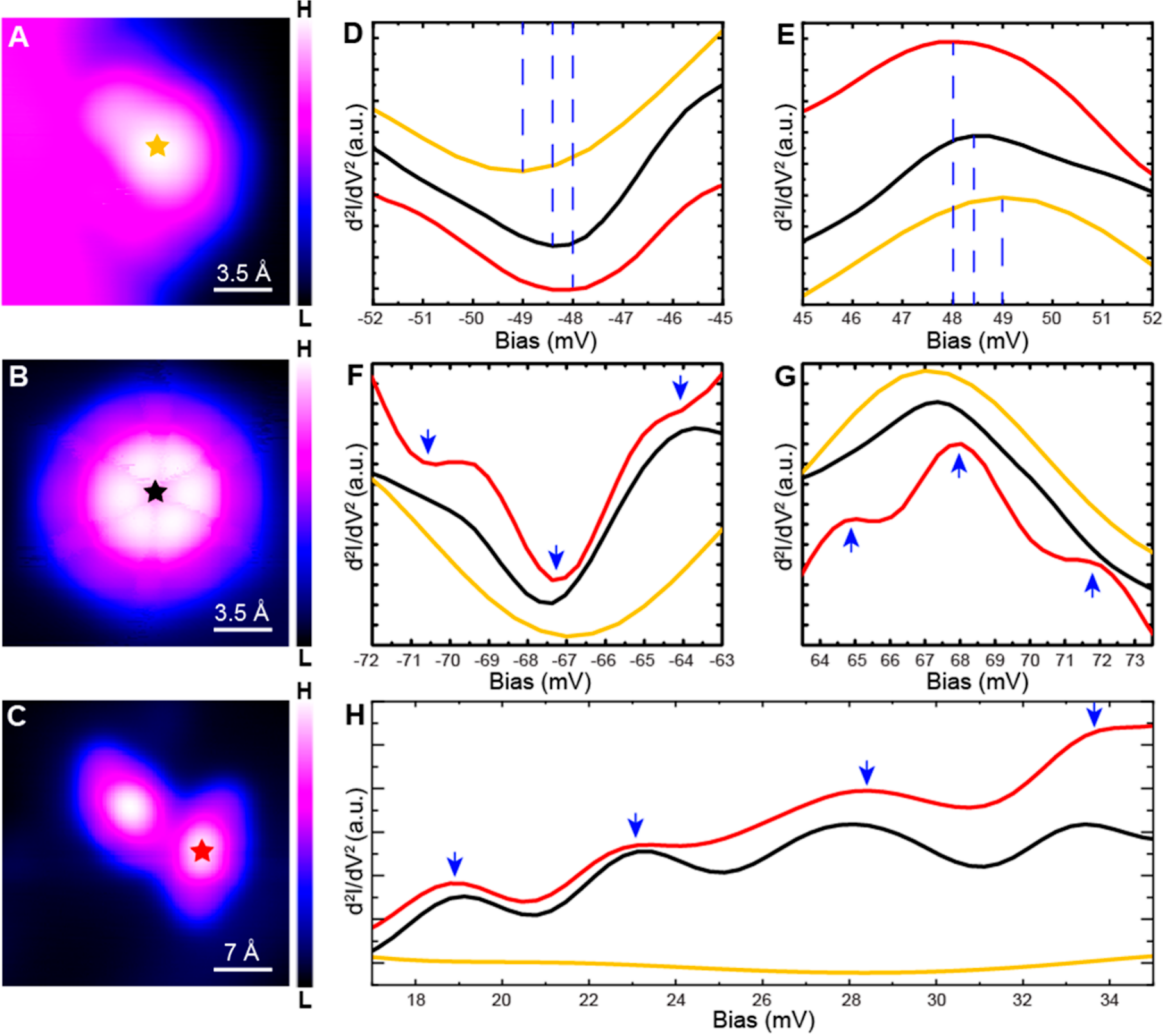
Effect of steric pressure and intermolecular interaction on molecular vibrations of CNAr^Mes2^. (A–C) Topographic images of CNAr^Mes2^ at the step edge (A), on the planar surface (B), and in a dimer configuration (C). (D–H) Comparison of IETS spectra demonstrating vibrational shifts (D,E), vibrational splitting (F,G), and vibrational quenching (H) of CNAr^Mes2^ ligands. The positions where the IETS spectra in (D–H) were collected are marked with orange, black, and red stars in (A–C). Imaging and spectroscopy parameters were set to −85 mV and 100 pA for (A,B), −500 mV and 30 pA for (C), and −85 mV and 1.2 nA for (D–H).

The energies of the vibrational modes we observed vary in response to the molecular adsorption at different sites (Figures 5A–C and S10A), highlighting the effects of steric pressure and interference on molecular properties. At both the step edge and herringbone elbow site, the bouncing vibration (mode V) shows a blue-shift compared to CNAr^Mes2^ ligands adsorbed on the basal plane (Figures 5D,E, and S10D,E), in good agreement with the theoretical simulation in Figure 4B. This vibration involves the stretching motion of the carbon–metal bond. The increase in vibrational energy is consistent with a strengthened carbon–metal interaction at both the step edge and the herringbone elbow site, resulting from the reduced out-of-plane steric repulsion between side arene groups and the metal substrate. It is important to note that the observed energy shift of mode V is unlikely to originate from varied tip-molecule interactions at different surface sites because mode V does not show detectable response to different tip-molecule distances (Figure S11). In contrast, modes below 35 meV are largely absent in the spectra taken over the molecule at the step edge (Figure 5H) as they involve frustrated rotation of the molecule in the polar direction and are likely quenched by the geometric limitation at the step edge. The simulated tunneling current captures the quenching of frustrated rotational modes I and III at the step edge, but the impact on modes II and IV is smaller compared with the experimental observation. We attribute this discrepancy to the selection rule of STM-IETS,^65,66^ which remains not fully understood.

A major difference between a molecule residing in a nanoenvironment and the ones in solution or a condensed phase arises from their intermolecular interactions. For *m*-terphenyl ligands, the intermolecular interaction is also a critical determinant of steric pressure. These implications include the subtle energetic balance between attractive ligand–ligand interactions (i.e., due to van der Waals forces) and the central attractive forces that dictate ligand–surface binding. However, precise imaging and spectroscopic observation of these intermolecular interactions are extremely rare, despite their significant implications for understanding chemical binding to surfaces. Notably, we have observed distinct modifications in the vibrational features resulting from ligand–ligand interactions. For instance, the formation of a dimer between two molecules (as depicted in Figure 5C) leads to an attractive interaction between the *para*-methyl group and the aryl ring of the adjacent molecule. Consequently, this interaction releases steric pressure on one side of the molecule while intensifying it on the other side, as shown in the theoretical simulation in Figure 4I,J. Experimentally, the redistribution of the steric pressure results in observed red-shifts in the bouncing mode V of the CNAr^Mes2^ ligands (Figure 5D,E), which can be attributed to a reduction in the ligand–surface bonding interaction as a consequence of strengthened ligand–ligand intermolecular interactions.^67^ More strikingly, a clear splitting of the methyl twisting mode VI is observed (Figure 5F,G), resulting from the asymmetric steric pressure applied on the different ortho-methyl groups in the interacting ligands. According to the theoretical simulation of the structure and strain presented in Figure 4I,J, it is observed that steric strain is alleviated in certain methyl groups while accentuated in others, leading to the lifting of energy degeneracy in the C–H bending motion due to intermolecular interactions between two molecules. Based on these results, it is likely that intermolecular interaction between ligands may diminish the strength of ligand–surface interactions, especially in low ligand-coverage scenarios.

## Conclusions

In summary, our study examined the influence of steric interference on the surface adsorption behavior of *m*-terphenyl isocyanide ligands using STM. The microscopic observations align with the rotational and vibrational dynamics of the most favorable molecular binding configurations predicted by MD simulations. Additionally, we evaluated the effect of steric pressure on molecular vibrational properties through IETS and computational simulation. Our results unambiguously show that the steric repulsion applied on the individual CNAr^Mes2^ ligands is reduced when adsorbed on a convex surface, leading to a site-selective molecular binding. The submolecular-scale characterization provides detailed insights into the unique chemical environment experienced by each individual ligand, revealing information that was previously inaccessible through ensemble measurements. Specifically, the vibrational characterization of (i) individual CNAr^Mes2^ ligands adsorbed on surface sites with varying curvatures and (ii) a ligand dimer reveals the influence of ligand–surface and intermolecular interactions on modifying the steric response of the constituent molecules. This information holds particular significance in the field of nanoscience, where even minor variations in the nanoscale chemical environment have been demonstrated to significantly impact molecular behavior.^68^

Overall, the unprecedented molecular-scale study of binding dynamics of a ligand using the steric encumbrance as a design principle on Au(111) brings us one step closer to deciphering real and nonideal ligand–surface systems. Moreover, the factors demonstrated to strongly influence the binding behavior of CNAr^Mes2^ have the potential to facilitate the rational design of other ligands targeting diverse surface topologies.

## Supporting Information Available

Details of experimental and computational methods and additional data (Figures S1 to S11 and Table S1). (PDF)
MD simulation of CNAr^Mes2^ on planar surface of Au(111) at 5 K. (MP4)
MD simulation of CNAr^Mes2^ on herringbone of Au(111) at 5 K. (MP4)
MD simulation of CNAr^Mes2^ at step edge of Au(111) at 5 K. (MP4)
Every second of the supplemental videos depicts approximately 25 picoseconds of simulated molecular dynamics occurring at the respective adsorption site.
