# Supplementary material for: Molecular-Scale Visualization of Steric Effects of Ligand Binding to Reconstructed Au(111) Surfaces
Source: J Am Chem Soc. 2024 Apr 16;146(17):11764–72. doi: 10.1021/jacs.4c00002 (PMC11066864; doi:10.1021/jacs.4c00002)
Supplement: Supplementary file 1 — ja4c00002_si_001.pdf [file ja4c00002_si_001.pdf]

Supporting Information

Molecular-Scale Visualization of Steric Effects of Ligand Binding to Reconstructed Au(111) Surfaces

Liya Bi[1,2], Sasawat Jamnuch[3], Amanda Chen[3], Alexandria Do[2,3], Krista P. Balto[1], Zhe Wang[4], Qingyi Zhu[1], Yufei Wang[2,3], Yanning Zhang[4], Andrea R. Tao[1,2,3]*, Tod A. Pascal[2,3]*, Joshua S. Figueroa[1,2]*, Shaowei Li[1,2]*

[1]Department of Chemistry and Biochemistry, University of California, San Diego, California 92093-0309, USA

[2]Program in Materials Science and Engineering, University of California, San Diego, California 92093-0418, USA

[3]Department of Nano and Chemical Engineering, University of California, San Diego, California 92093-0448, USA

[4]Institute of Fundamental and Frontier Sciences, University of Electronic Science and Technology of China, Chengdu 611731, China.

*Corresponding authors. Email: atao@ucsd.edu (Andrea R. Tao); tpascal@ucsd.edu (Tod A. Pascal); jsfig@ucsd.edu (Joshua S. Figueroa); shaoweili@ucsd.edu (Shaowei Li)

1. Experimental methods

The STM and IETS measurements were performed with a customized CreaTec low-temperature STM operating at ~ 5 K and a base pressure of < 1×10^{-10} Torr. The Au(111) substrate was cleaned by cycles of successive Ar^+ sputtering and thermal annealing at around 870 K. The electrochemically etched W tip was first cleaned and sharpened by Ar^+ sputtering and thermal annealing, and further conditioned by repeatedly poking on the Au(111) surface until single-molecule resolution was achieved. The $CNAr^{Mes2}$ ligands were synthesized and characterized according to previously published procedures.[1] The ambient thermogravimetric analysis (TGA) plot of $CNAr^{Mes2}$ powder (**Figure S1A**) shows an onset evaporation temperature of ~ 170 °C and no decomposition into solid residues. We therefore dosed the $CNAr^{Mes2}$ ligands onto the clean Au(111) surface at ~ 5 K by thermal sublimation with a homemade Knudsen cell evaporator in the vacuum chamber. The $CNAr^{Mes2}$ on Au(111) distinguishes itself from other surface-adsorbed impurities by its much larger apparent height and its distinct contour (**Figure S2)**. To exam the thermal induced dynamics of $CNAr^{Mes2}$ ligands on Au(111), the sample was gradually warmed from the 5 K at the STM junction to room temperature by removing the sample from the STM cryostat. Subsequently, the sample was cooled down to 5 K again for surface examination. (**Figure 1E and Figures S2B, C, E**).

The topographic images were all taken under the constant current mode by recording z with the feedback on and were processed with Gwyddion[2]. The d^2I/dV^2 spectra were acquired by numerically differentiating the first harmonic output (dI/dV) of the lock-in amplifier. The modulation on the sample bias was 2-3 mV (root mean square) at a frequency of 933 Hz and the feedback was off while sweeping the bias. The d^2I/dV^2 images were taken in a way similar to constant-current topographic imaging (z). At each pixel in the image, the second harmonic of tunneling current (d^2I/dV^2) was recorded for a chosen bias with the feedback turned off. A 7 mV (RMS) and 511 Hz modulation was applied on the sample bias. The feedback was turned on again before collecting signal at the next pixel to set same tip-sample distance/interaction.

2. Computational methods

a. Quantum Mechanics (QM) calculations of $CNAr^{Mes2}$ binding to Au surfaces

Au slabs, representing the planar, herringbone and step-edge geometries (**Figure S6**), were constructed from the Au FCC crystal (4 × 4 × 4 supercell). The z-coordinate of the unit cell was inflated by at least 2 nm of vacuum, in order to prevent spurious interactions with the neighboring cell. We then calculated the binding energy of a rigid $CNAr^{Mes2}$ ligand, by performing a rigid coordinate scan along the z-axis, where we solved for the electron density and thus energy of the system using Density Functional Theory (DFT), as implemented in the Quantum Espresso[3, 4] electronic structure package. These calculations employed ultrasoft pseudopotentials, a kinetic energy cutoff of 30 Ry, an energy density cutoff of 300 Ry and a (5,5,1) K-point grid.

b. Force Field Molecular Dynamics (FFMD) simulations of $CNAr^{Mes2}$ ligand dynamics on Au surfaces

We assessed the rotational dynamics of $CNAr^{Mes2}$ ligands at different sites on Au surface, by means of molecular dynamics (MD) simulations, where the energy of the system was obtained from analytic interaction potentials (i.e., forcefields). The simulated motions of the molecule are visualized in supplementary **Movies S1-S3**. Specifically, the Au – Au interactions were described using the many-body Embedded Atom Method, as described in the forcefield of Ackland,[5] while the valence and non-bonded (van der Waals) interactions of the $CNAr^{Mes2}$ were described using

UFF,[6] with Mulliken charges. The Au – $CNAr^{Mes2}$ van der Waals interactions were obtained from Lorentz-Berthelot[7] mixing rules, assuming the Au van der Waals parameters in UFF. We separately considered the $CNAr^{Mes2}$ carbon – Au interactions, by fitting the binding energy curves of the $CNAr^{Mes2}$ interacting with the Au(111) surfaces from QM (part above) to a Morse potential.[8]

The van der Waals and real space coulomb cutoffs in the MD simulations were 10 Å. A cubic spline was applied to the van der Waals to ensure smooth convergence and vanishing energies and forces at the cutoff (inner cutoff distance of 9 Å). The reciprocal space coulomb interactions were computed with a particle-particle-particle-mesh solver, with an error tolerance of 10^{-6}.[9] Each MD simulation was initiated with 500 conjugated gradient steps, followed by gradual heating to 5 K using 0.5 ns (500,000 steps with an integration timestep of 1 fs) dynamics in the canonical ensemble (NVT – constant number of particles N, volume V and temperature T = 5 K). A Nose-Hoover thermostat was used with a temperature relaxation window of 100 fs. The time-reversible measure-preserving Verlet integrators derived by Tuckerman et al.[10] was applied for the time integration. After density equilibration, the system was simulated in the NVT ensemble for at least 5 ns of NVT dynamics. Starting with snapshots of each system every 1 ns, additional 40 ps NVT simulation were run, and the velocities and coordinates were saved every 4 fs (10,000 frames in the corresponding trajectory).

c. Rotational barrier of $CNAr^{Mes2}$ ligand on Au surfaces

We quantified the rotational characteristic of individual $CNAr^{Mes2}$ ligand on the three different sites on Au(111) by calculating the characteristic rotational temperature Θ_R, an estimate of the spacing between the rotational energy levels and thus, indicative of the temperature at which the 1st rotational energy level of the system is thermally populated:

$$\Theta_R = \frac{\hbar}{2k_B \langle I \rangle}$$

where I is the molecular moment of inertia and the angled brackets represent the statistical average from our MD simulations. We further resolve Θ_R into its in-plane (average of the x- and y-components of the I vector) and out-of-plane (z-component) contributions in **Table S1**. We found that the barrier for in-plane rotation of $CNAr^{Mes2}$ ligand on the planar surface is significantly smaller than in either the step edge or herringbone.

d. Vibrational properties of free $CNAr^{Mes2}$ and $CNAr^{Mes2}$ on Au surfaces

The vibrational density in **Figure 4A** were calculated with DFT as implemented in the Vienna ab initio simulation package (VASP)[11, 12] with the projector augmented wave (PAW) method.[13] The exchange-correlation functional was described by the generalized gradient approximation (GGA) in the form proposed by Perdew-Burke-Ernzerhof.[14, 15] The Au(111) surface was simulated by an 8 × 8 × 1 supercell model with a thickness of 3 layers. An energy cutoff of 550 eV was used for the plane-wave basis expansion, and a Γ-centered Monkhorst-Pack k-point mesh was adopted for the Brillouin-zone integration. The van der Waals interaction, which plays a fundamental role in physical adsorption processes, was corrected using the DFT-D3 method[16] with the Becke-Johnson damping function[17]. The vibrational frequencies and normal modes were calculated using the finite differences approach, and then a Lorentzian broadening of 1 eV was applied to yield the density of vibrational modes.

e. Non-Equilibrium Green's Function (NEGF) I/V curve simulation

To understand the change in tunneling current due to the vibrational excitation of $CNAr^{Mes2}$ ligand at different sites, we performed electron-transport calculations using the numerical atomic orbital DFT code SIESTA[18, 19] using the PBE exchange-correlation functional in **Figure 4B**.[15] A mesh cutoff defining the real-space grid of 500 Ry was used, while the electronic structure was modelled using a double-ζ with polarization (DZP) basis set. The transport properties were then calculated using a NEGF approach as implemented in the TRANSIESTA code.[20] Here, simulation cells comprising a top Au(111) electrode, a 8 × 4 × 6 supercell, and the optimized $CNAr^{Mes2}$ structure on various Au surfaces (the bottom electrode with herringbone, step edge and planar morphologies) was used. The active region on both electrodes was taken as the 4 layers closest to the $CNAr^{Mes2}$ molecule (see **Figure S9**). If inelastic tunneling processes occur at the STM junction, the tunneling current comprises two components: elastic tunneling current I_{el} and the inelastic tunneling current I_{ie}. The total tunneling current can be written as[21, 22]:

$$I(V_b) = G_0 \int_{\mu_R}^{\mu_L} T(E,V_b)\theta(V_b - h\nu/e)dE$$

where $\theta\left(V_b - \frac{h\nu}{e}\right)$ is a step function that reflects the conductance increase at a vibrational frequency ν due to the opening of an inelastic tunneling channel, $G_0 = 2(e^2/h)$ is a constant (unit of quantum conductance), and $T(E, V_b)$ is the elastic electron tunneling probability through the junction under a bias V_b. In some earlier studies, the $T(E, V_b)$ term is considered as a slow varying function and treated as a constant.[21] This approximation underestimates the contribution from the elastic tunneling channel to the signal. Here, we obtain $T(E, V_b)$ through DFT simulation, including the contribution from nucleus motions. Due to the molecular vibrations, the elastic tunneling channel gives current peaks at the vibrational thresholds. To determine the vibration energies, we may focus only on the elastic tunneling current using the Landauer-Buttiker formula[23]:

$$I_{el}(V_b) = G_0 \int_{\mu_R}^{\mu_L} T(E,V_b)dE$$

The peak positions in the simulated I_{el} coincide with different vibrational energies. The voltage bias between the two electrodes was then varied, from 0 to 100 meV in increments of 1 meV to generate the I/V curve.

f. Evaluation of the strain distribution of $CNAr^{Mes2}$ dimer

The strain energies of the $CNAr^{Mes2}$ dimer in Figures 4I-J were obtained by MD simulations at 5 K, considering the difference between the energy per atom in the dimer, and that of the isolated monomer. The energy per atom was averaged over our 40 ps NVT simulation and includes contributions from the valence (energies related to displacement from equilibrium of the bonds, angles and torsions), the van der Waals and electrostatic energy contributions.

Supplementary Text

<u>Section 1. Steric-pressure induced adsorption instability of individual $CNAr^{Mes2}$ ligand on herringbone elbow sites</u>

We found that the adsorption of $CNAr^{Mes2}$ ligand is metastable on the herringbone elbow site. In other words, its adsorption can be easily perturbed. **Figure S4D** shows that the individual $CNAr^{Mes2}$ ligand in **Figure S4C** switches to another adsorption configuration during scanning.

Figure S4F shows that the $CNAr^{Mes2}$ ligand in **Figure S4E** switches between two adsorption geometries when scanned by a closer tip. In combination with the observation of highly stable $CNAr^{Mes2}$ ligand at the step edge (**Figure 2B and Figure S2E**) and constantly rotating molecule on planar surface (**Figure 2C and Figure S5**), we concluded that the steric pressure between the mesityl groups and substrate mediates molecular adsorption and makes it preferential to bind at the step edge, but less stable to bind on herringbone elbow site, and nearly freely rotating on planar surface. Additionally, even though $CNAr^{Mes2}$ forms clusters on the FCC domain, this adsorption configuration remains metastable. Individual molecules in the cluster can be readily manipulated by the tip (**Figures S4A,B**). This suggests that the intermolecular interactions among $CNAr^{Mes2}$ are insufficient to fully stabilize the binding of individual molecules on the planar surface.

Section 2. DFT calculated vibrational modes of $CNAr^{Mes2}$ ligands

The calculated vibration energies of $CNAr^{Mes2}$ ligands on Au(111) (**Figure S8**) match well with those observed experimentally (**Figure 3**). The DFT results also show that upon adsorption on Au(111), the vibration energy of $CNAr^{Mes2}$ ligands shifts with respect to that of the free molecules due to the ligand-surface interaction, which is supported by our experimental finding that both intermolecular interaction and steric repulsion between $CNAr^{Mes2}$ ligand and Au(111) can modify molecular vibrations (**Figure 5**). It's noteworthy that the experimentally resolved vibrational modes (IETS peaks/dips) could be broadened by several adjacent molecular vibrations, as suggested by the d^2I/dV^2 mapping of $CNAr^{Mes2}$ at 33.4 mV (**Figure 3E**) and the simulation of vibrational modes of $CNAr^{Mes2}$ at nearby energy (**Figure 4F**).

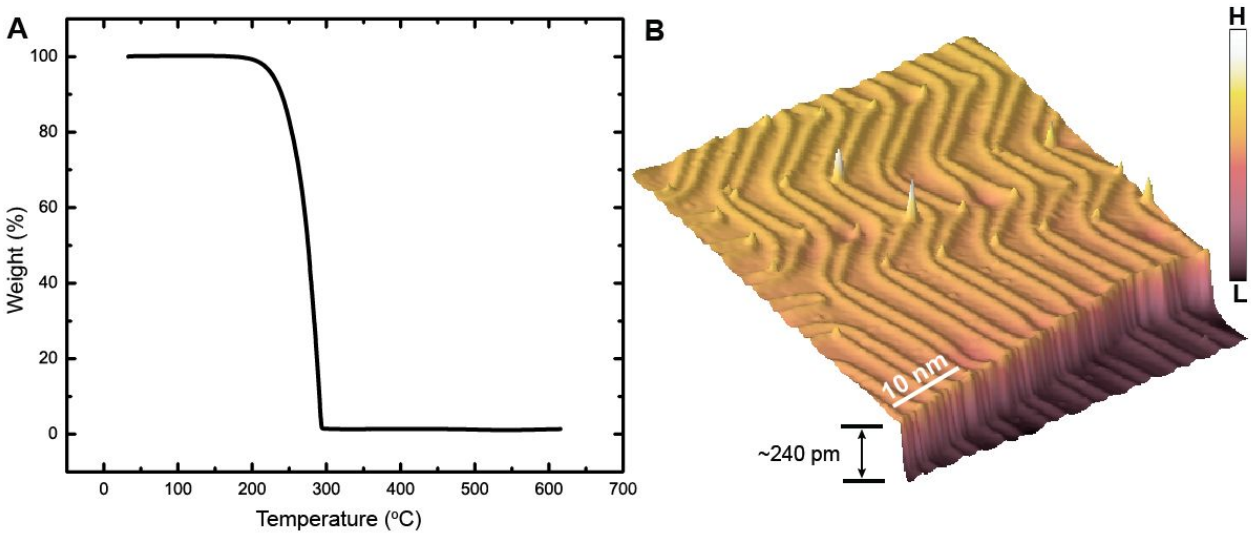

Figure S1.

(**A**) Ambient TGA plot of $CNAr^{Mes2}$ powder shows a ~ 170 °C onset evaporation temperature. (**B**) 3D view of Au(111) shows the diverse surface structures including the step edges, herringbones and flat domains between herringbones, together with a low density of impurities. Imaging setpoint: -1.5 V, 100 pA.

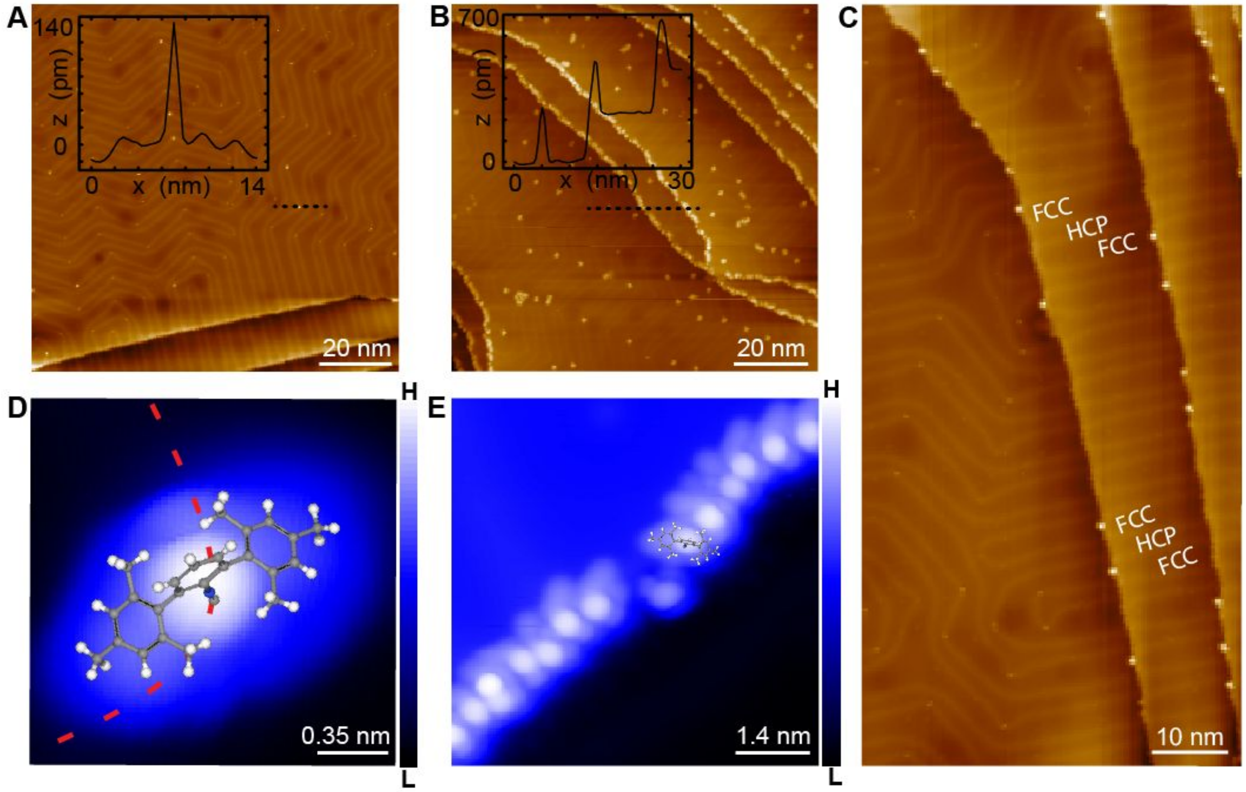

Figure S2.

Topographic signature of $CNAr^{Mes2}$ ligands on Au(111). (**A**) Au(111) without $CNAr^{Mes2}$ ligands. The inset is the linecut across the black dashed line, showing ~ 140 pm apparent height of an impurity at herringbone elbow site. (**B**) Au(111) with $CNAr^{Mes2}$ ligands adsorbed at the step edges, herringbone elbow sites and clustered on FCC basal planes. The inset shows the landscape across both the $CNAr^{Mes2}$ on herringbone elbow site and at the step edges. The apparent height of $CNAr^{Mes2}$ ligands is much larger than that of the impurities on herringbone elbow sites. (**C**) Topographic image of the surface with low-density $CNAr^{Mes2}$ after warming up to room temperature and re-cooling to 5 K. The FCC and HCP domains are labeled for clarity. (**D**) High-resolution image of a single $CNAr^{Mes2}$ on herringbone elbow site acquired with a molecular tip. The red dashed curve indicates the herringbone. The overlaid model is scaled to the image size and is for visualizing the adsorption geometry only. The N, C and H atoms are shown in blue, grey and white respectively. (**E**) High-resolution image of $CNAr^{Mes2}$ ligands at Au(111) step edge acquired with a molecular tip. The scaled molecular model is superimposed on a $CNAr^{Mes2}$ and is for visualizing the adsorption geometry only. Setpoint: -1.5 V, 100 pA (A); 1 V, 100 pA (B); -1 V, 50 pA (C); -610 mV, 50 pA (D); -1 V, 100 pA (E).

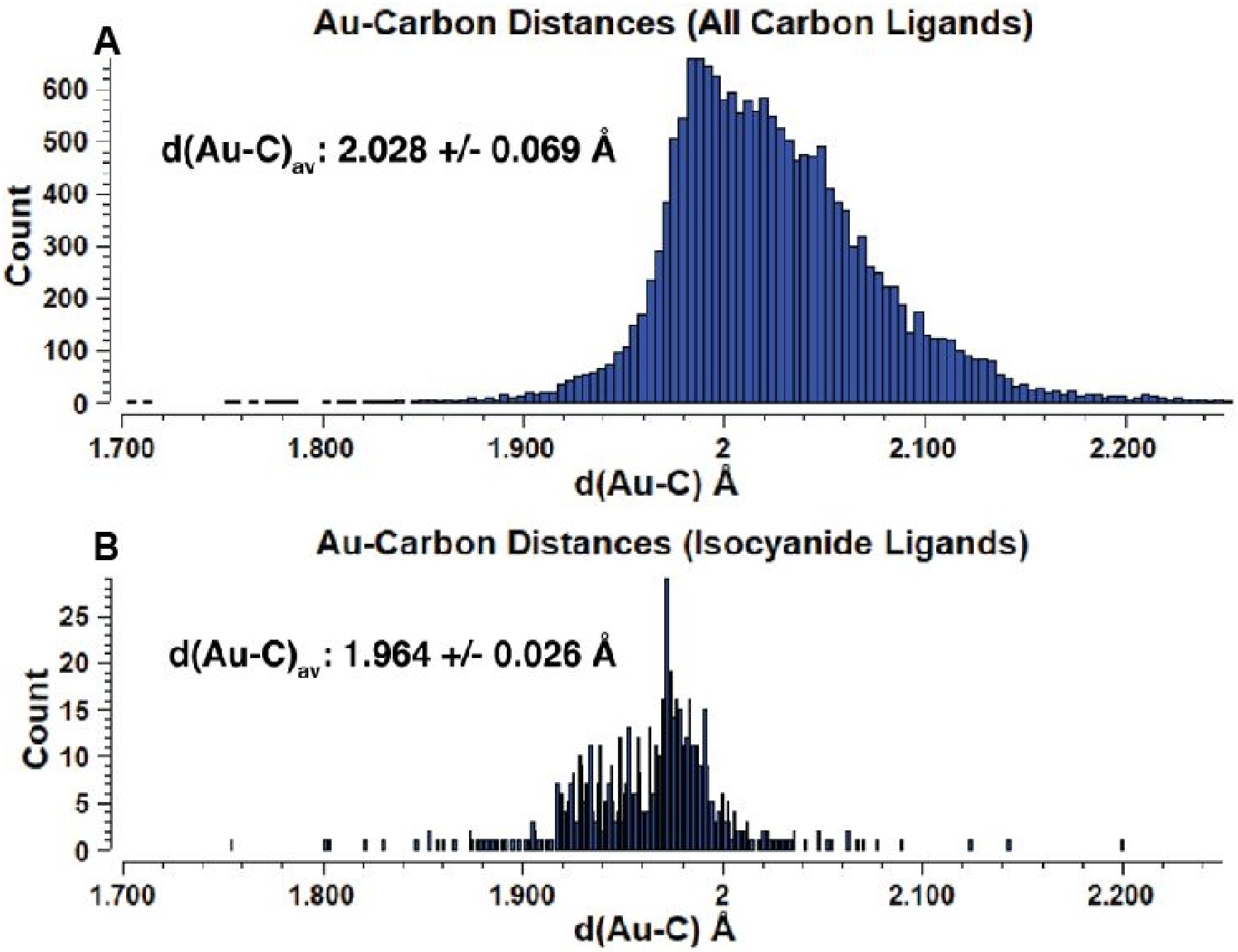

Figure S3.

Cambridge Structural Database[24] Au-C bond distances for structurally characterized molecular Au complexes with carbon-based ligands. Note that all gold oxidation states are represented. (**A**) Histogram of Au-C bond distances for structurally characterized molecular gold complexes with any carbon-based ligand. The average Au-C bond distance is 2.028 +/- 0.069 Å. (**B**) Histogram of Au-C bond distances for structurally characterized molecular gold complexes limited to isocyanide (C≡NR) ligands. The average Au-C bond distance is 1.964 +/- 0.026 Å, which corresponds well with the results of computational characterization of $CNAr^{Mes2}$ ligands at Au edge (**Figure 1G**).

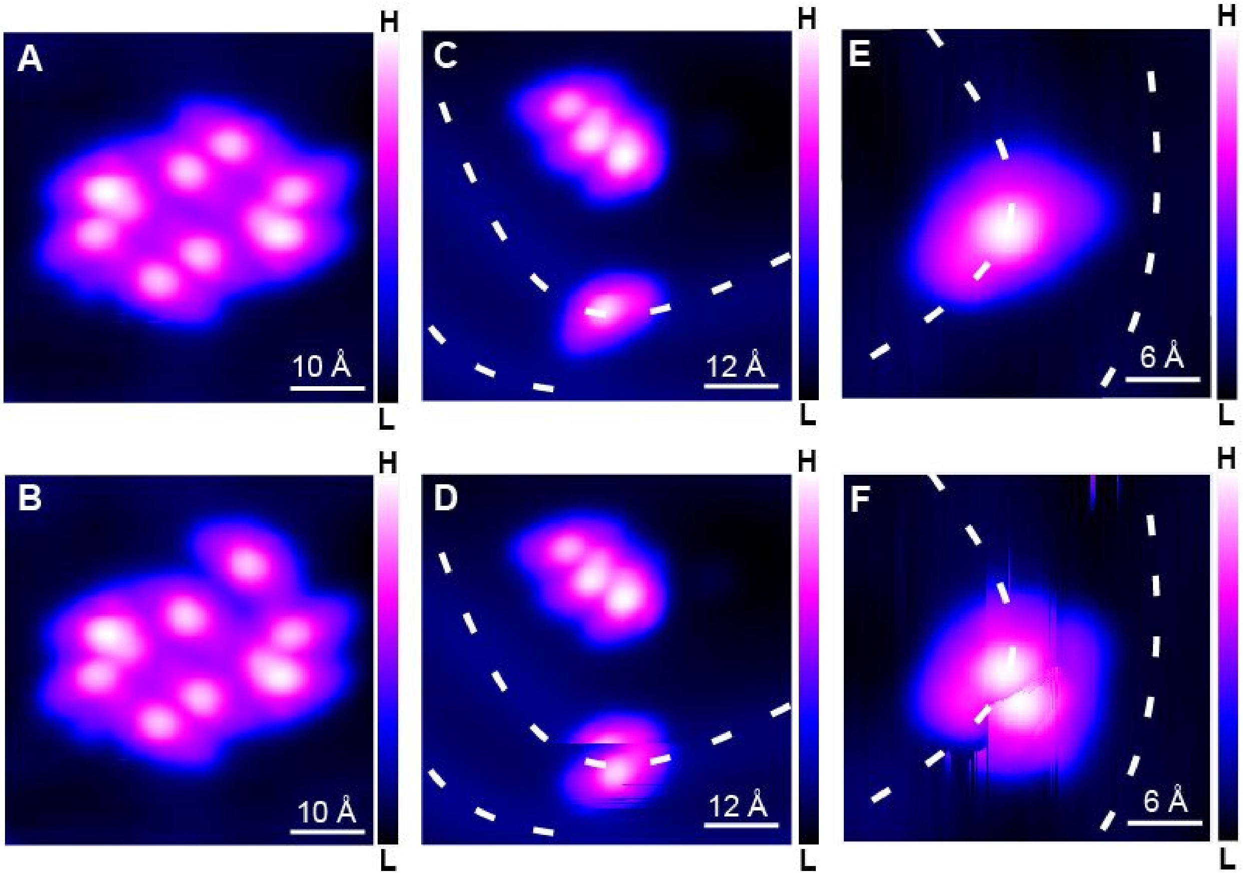

Figure S4.

The movement of single CNAr^{Mes2} ligand induced by tip perturbation or tunneling electrons. (**A**) Topographic image of a CNAr^{Mes2} cluster on the FCC domain. Setpoint: -50 mV, 20 pA. (**B**) Topographic image of the same cluster in (A) right after being perturbed by a closer tip-sample distance (perturbation tunneling gap set at -10 mV, 50 pA). One CNAr^{Mes2} slightly moved away from the molecular cluster. Imaging setpoint: -50 mV, 20 pA. (**C and D**) Topographic image of a CNAr^{Mes2} cluster on the FCC domain and a CNAr^{Mes2} on herringbone elbow site at the same setpoint: -1 V, 100 pA, indicating the excitation of CNAr^{Mes2} by tunneling electrons. (**E**) Topographic image of static CNAr^{Mes2} ligand on the herringbone elbow site. Setpoint: -50 mV, 20 pA. (**F**) Topographic image of the same molecule in (E) but at a closer tip-sample distance. Setpoint: -10 mV, 50 pA. The white dashed curves in (C)-(F) indicate the herringbones.

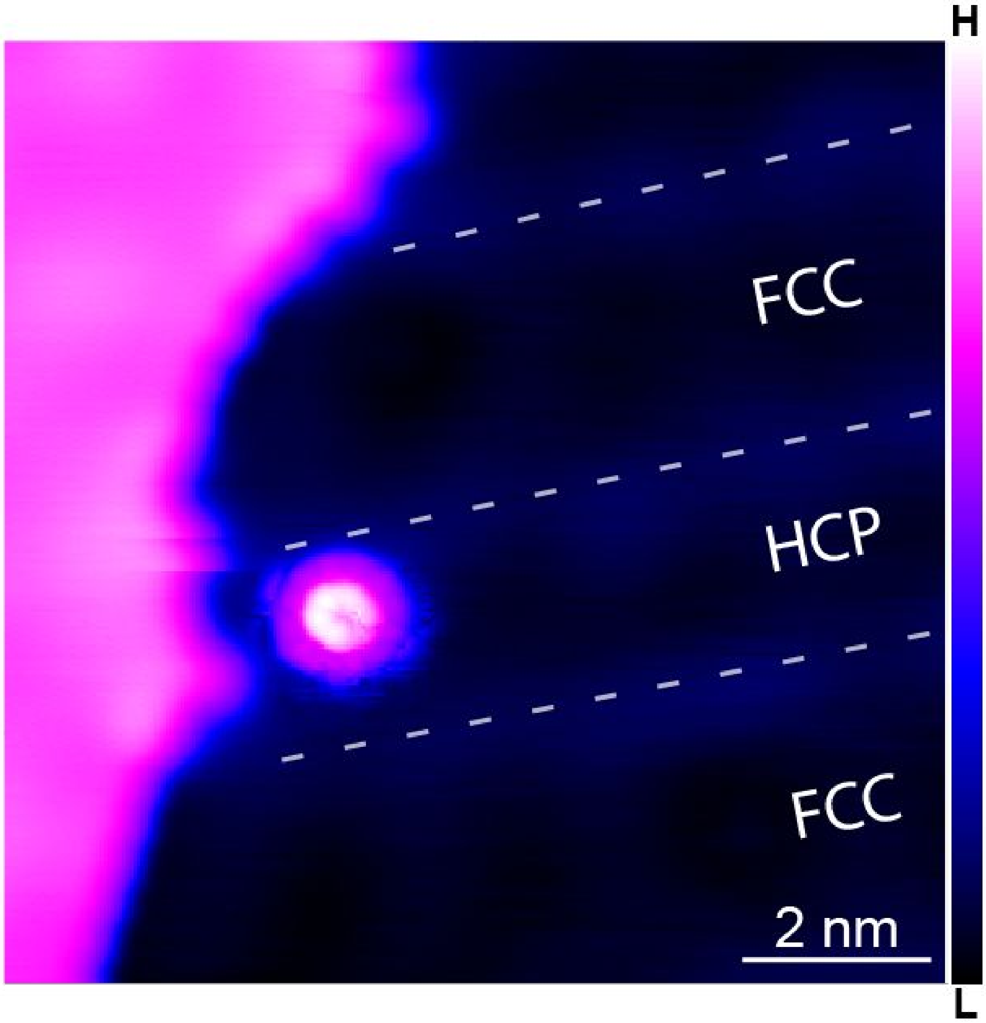

Figure S5.

Topographic image of a constantly rotating CNAr^{Mes2} ligand on the HCP basal plane. The white dashed curves indicate the herringbones. HCP and FCC domains are labeled for clarity. Setpoint: -85 mV, 100 pA.

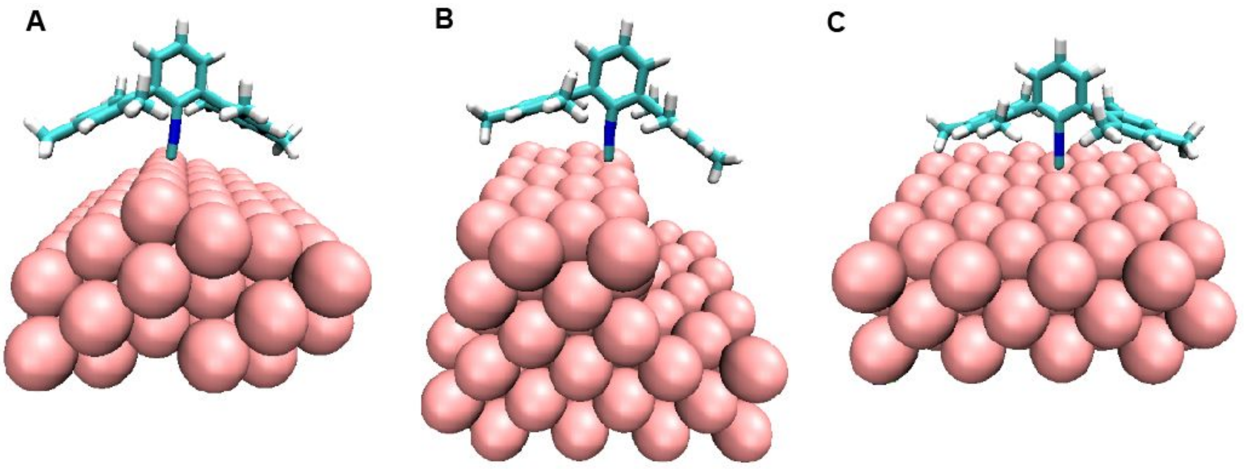

Figure S6.

Simulated adsorption geometries of individual $CNAr^{Mes2}$ ligand at different sites on Au(111). (**A**) On herringbone elbow site. (**B**) At the step edge. (**C**) On planar surface.

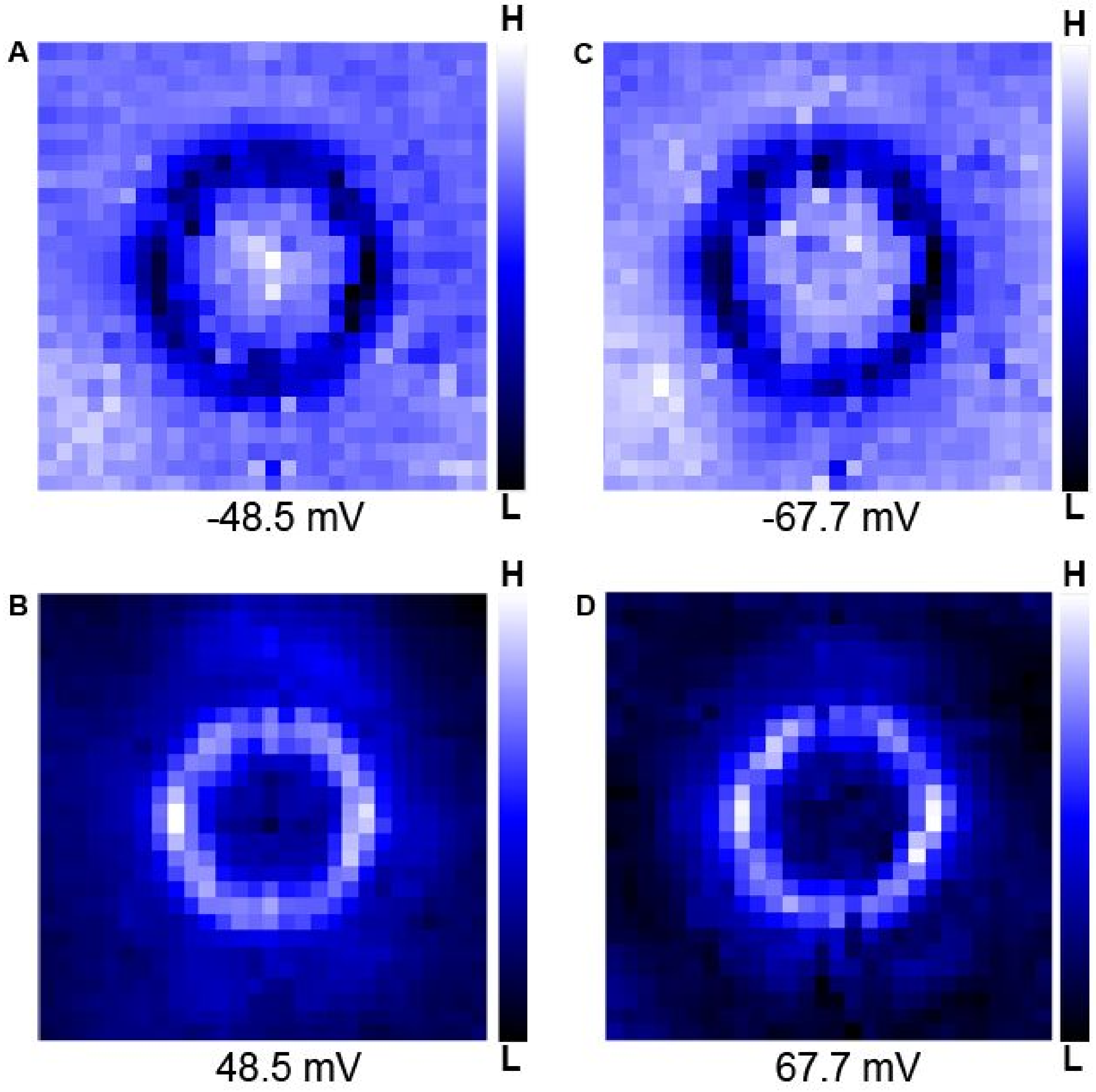

Figure S7.
***d^2I/dV^2* mappings of the rotating $CNAr^{Mes2}$ ligand at ± 48.5 mV (A and B) and ± 67.7 mV (C and D). Setpoint: -67.7 mV, 400 pA.**

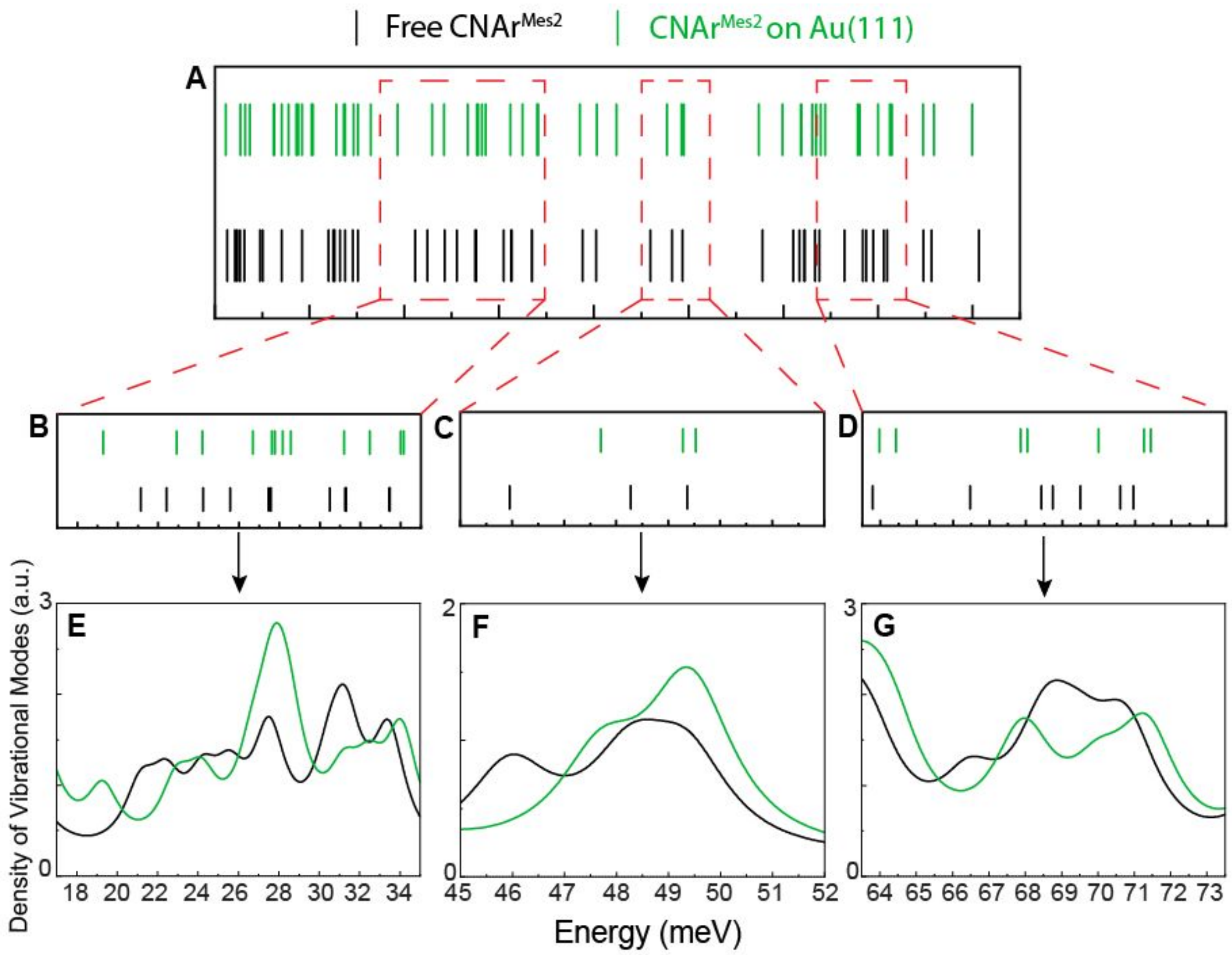

Figure S8.

DFT calculation of vibration energy (A)-(D) and density of vibrational modes (E)-(G) of free (black) and on-surface (green) CNAr^{Mes2} ligands.

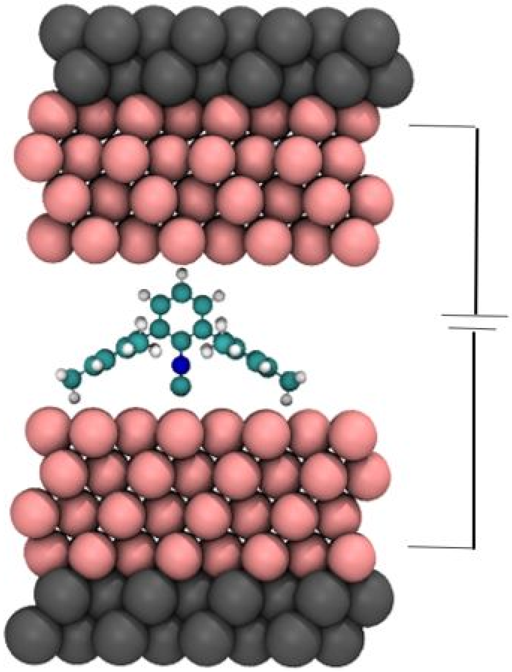

Figure S9.
Schematic of simulation cell setup for simulating the I/V curve of a CNAr^{Mes2} ligand on a planar surface. The active Au regions are shown in pink balls and the tunneling probabilities calculated for transmission of an electron from the top to the bottom electrode. The black atoms are inactive and act as an internal reference.

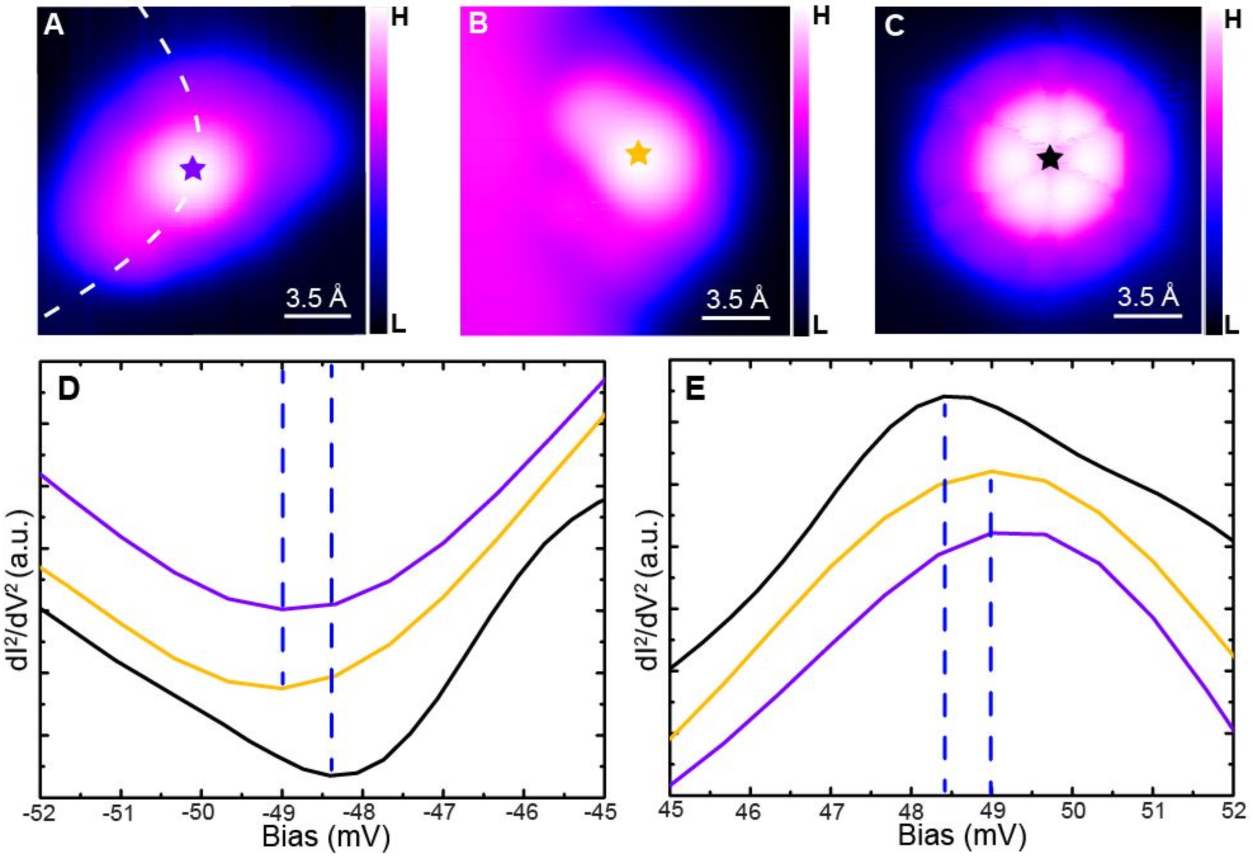

Figure S10.

Comparison on mode V among CNAr^{Mes2} at different surface sites. (**A**)-(**C**) Topographic images of CNAr^{Mes2} on the herringbone elbow site (A), at the step edge (B), and on the planar surface (C). Setpoint: -50 mV, 20 pA (A); -85 mV, 100 pA (B and C). (**D** and **E**) Comparison on mode V, the bouncing vibration, among CNAr^{Mes2} in (A)-(C). The positions where the IETS spectra were collected are marked with purple (-85 mV 500 pA), orange (-85 mV 1.2 nA), and black (-85 mV 1.2 nA) stars in (A)-(C). The IETS spectrum on the CNAr^{Mes2} on herringbone elbow site was taken with a larger tunneling gap to prevent switching of the molecule during spectroscopy.

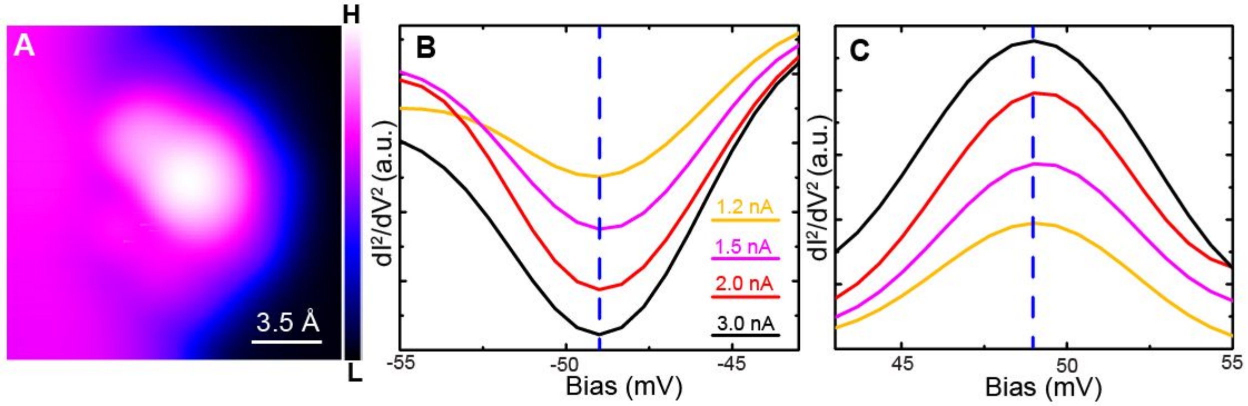

Figure S11.

Response of CNAr^{Mes2} mode V at step edge to different tip-molecule distances. (**A**) Topographic images of CNAr^{Mes2} at the step edge. Setpoint: -85 mV, 100 pA. (**B** and **C**) z-dependent measurements of the bouncing vibration, mode V, of CNAr^{Mes2} in (A). The IETS setpoint bias was -85 mV. The setpoint current is labeled in the graph. Although it has been reported that both the electric[25] and force[26] field change at the STM junction can alter the molecular vibrational energies, no detectable shift of mode V energy to these changes is observed here.

Table S1.

Spacing between energy levels (K) of $CNAr^{Mes2}$ ligands at different sites on Au(111). In-plane values are the average of the x and y components. Numbers in brackets indicate the uncertainty (standard deviation - 1σ)

**Au(111)**	**Rotational Temperatures (K)**			
	**x**	**y**	**z**	**In-plane**
**Herringbone**	5.00 (0.02)	5.09 (0.02)	2.22 (0.10)	5.05
**Planar**	2.81 (0.05)	2.96 (0.10)	2.3 (0.12)	2.90
**Step edge**	5.14 (0.03)	5.19 (0.04)	2.1 (0.09)	5.16

Movie S1.

MD simulation of $CNAr^{Mes2}$ on planar surface of Au(111) at 5 K

Movie S2.

MD simulation of $CNAr^{Mes2}$ on herringbone of Au(111) at 5 K

Movie S3.

MD simulation of $CNAr^{Mes2}$ at step edge of Au(111) at 5 K

Every second in the supplemental videos captures ~25 ps of molecular dynamics simulated at the corresponding adsorption site.

References

1. Fox, B. J.; Sun, Q. Y.; DiPasquale, A. G.; Fox, A. R.; Rheingold, A. L.; Figueroa, J. S., Solution Behavior and Structural Properties of Cu(I) Complexes Featuring m-Terphenyl Isocyanides. *Inorganic Chemistry* **2008,** *47* (19), 9010-9020.
2. Nečas, D.; Klapetek, P., Gwyddion: an open-source software for SPM data analysis. **2012,** *10* (1), 181-188.
3. Giannozzi, P.; Baroni, S.; Bonini, N.; Calandra, M.; Car, R.; Cavazzoni, C.; Ceresoli, D.; Chiarotti, G. L.; Cococcioni, M.; Dabo, I.; Corso, A. D.; Gironcoli, S. d.; Fabris, S.; Fratesi, G.; Gebauer, R.; Gerstmann, U.; Gougoussis, C.; Kokalj, A.; Lazzeri, M.; Martin-Samos, L.; Marzari, N.; Mauri, F.; Mazzarello, R.; Paolini, S.; Pasquarello, A.; Paulatto, L.; Sbraccia, C.; Scandolo, S.; Sclauzero, G.; Seitsonen, A. P.; Smogunov, A.; Umari, P.; Wentzcovitch, R. M., QUANTUM ESPRESSO: a modular and open-source software project for quantum simulations of materials. *J. Phys.: Condens. Matter* **2009,** *21*, 395502.
4. Giannozzi, P.; Andreussi, O.; Brumme, T.; Bunau, O.; Buongiorno Nardelli, M.; Calandra, M.; Car, R.; Cavazzoni, C.; Ceresoli, D.; Cococcioni, M.; Colonna, N.; Carnimeo, I.; Dal Corso, A.; de Gironcoli, S.; Delugas, P.; DiStasio, R. A.; Ferretti, A.; Floris, A.; Fratesi, G.; Fugallo, G.; Gebauer, R.; Gerstmann, U.; Giustino, F.; Gorni, T.; Jia, J.; Kawamura, M.; Ko, H. Y.; Kokalj, A.; Küçükbenli, E.; Lazzeri, M.; Marsili, M.; Marzari, N.; Mauri, F.; Nguyen, N. L.; Nguyen, H. V.; Otero-de-la-Roza, A.; Paulatto, L.; Poncé, S.; Rocca, D.; Sabatini, R.; Santra, B.; Schlipf, M.; Seitsonen, A. P.; Smogunov, A.; Timrov, I.; Thonhauser, T.; Umari, P.; Vast, N.; Wu, X.; Baroni, S., Advanced capabilities for materials modelling with Quantum ESPRESSO. *Journal of Physics: Condensed Matter* **2017,** *29* (46), 465901.
5. Ackland, G. J.; Tichy, G.; Vitek, V.; Finnis, M. W., Simple N-body potentials for the noble metals and nickel. *Philosophical Magazine A* **1987,** *56* (6), 735-756.
6. Rappe, A. K.; Casewit, C. J.; Colwell, K. S.; Goddard, W. A., III; Skiff, W. M., UFF, a full periodic table force field for molecular mechanics and molecular dynamics simulations. *Journal of the American Chemical Society* **1992,** *114* (25), 10024-10035.
7. Lorentz, H. A., Ueber die Anwendung des Satzes vom Virial in der kinetischen Theorie der Gase. *Annalen der Physik* **1881,** *248* (1), 127-136.
8. Wang, Y.; Chen, A. A.; Balto, K. P.; Xie, Y.; Figueroa, J. S.; Pascal, T. A.; Tao, A. R., Curvature-Selective Nanocrystal Surface Ligation Using Sterically-Encumbered Metal-Coordinating Ligands. *ACS Nano* **2022,** *16* (8), 12747-12754.
9. Hockney, R. W.; Eastwood, J. W., *Computer simulation using particles*. Adam Hilger: NY, 1989.
10. Tuckerman, M. E.; Alejandre, J.; López-Rendón, R.; Jochim, A. L.; Martyna, G. J., A Liouville-operator derived measure-preserving integrator for molecular dynamics simulations in the isothermal–isobaric ensemble. *Journal of Physics A: Mathematical and General* **2006,** *39* (19), 5629.
11. Kresse, G.; Furthmüller, J., Efficient iterative schemes for ab initio total-energy calculations using

a plane-wave basis set. *Physical Review B* **1996,** *54* (16), 11169-11186.

12. Kresse, G.; Hafner, J., Ab initio molecular-dynamics simulation of the liquid-metal--amorphous-semiconductor transition in germanium. *Physical Review B* **1994,** *49* (20), 14251-14269.

13. Blöchl, P. E., Projector augmented-wave method. *Physical Review B* **1994,** *50* (24), 17953-17979.

14. Perdew, J. P.; Burke, K.; Wang, Y., Generalized gradient approximation for the exchange-correlation hole of a many-electron system. *Physical Review B* **1996,** *54* (23), 16533-16539.

15. Perdew, J. P.; Burke, K.; Ernzerhof, M., Generalized Gradient Approximation Made Simple. *Phys Rev Lett* **1996,** *77* (18), 3865-3868.

16. Grimme, S.; Antony, J.; Ehrlich, S.; Krieg, H., A consistent and accurate ab initio parametrization of density functional dispersion correction (DFT-D) for the 94 elements H-Pu. *The Journal of Chemical Physics* **2010,** *132* (15).

17. Grimme, S.; Ehrlich, S.; Goerigk, L., Effect of the damping function in dispersion corrected density functional theory. *Journal of Computational Chemistry* **2011,** *32* (7), 1456-1465.

18. García, A.; Papior, N.; Akhtar, A.; Artacho, E.; Blum, V.; Bosoni, E.; Brandimarte, P.; Brandbyge, M.; Cerdá, J. I.; Corsetti, F.; Cuadrado, R.; Dikan, V.; Ferrer, J.; Gale, J.; García-Fernández, P.; García-Suárez, V. M.; García, S.; Huhs, G.; Illera, S.; Korytár, R.; Koval, P.; Lebedeva, I.; Lin, L.; López-Tarifa, P.; Mayo, S. G.; Mohr, S.; Ordejón, P.; Postnikov, A.; Pouillon, Y.; Pruneda, M.; Robles, R.; Sánchez-Portal, D.; Soler, J. M.; Ullah, R.; Yu, V. W.-z.; Junquera, J., Siesta: Recent developments and applications. *The Journal of Chemical Physics* **2020,** *152* (20).

19. José, M. S.; Emilio, A.; Julian, D. G.; Alberto, G.; Javier, J.; Pablo, O.; Daniel, S.-P., The SIESTA method for ab initio order-N materials simulation. *Journal of Physics: Condensed Matter* **2002,** *14* (11), 2745.

20. Papior, N.; Lorente, N.; Frederiksen, T.; García, A.; Brandbyge, M., Improvements on non-equilibrium and transport Green function techniques: The next-generation transiesta. *Computer Physics Communications* **2017,** *212*, 8-24.

21. Lambe, J.; McCarthy, S. L., Light Emission from Inelastic Electron Tunneling. *Physical Review Letters* **1976,** *37* (14), 923-925.

22. Persson, B. N. J.; Baratoff, A., Theory of photon emission in electron tunneling to metallic particles. *Physical Review Letters* **1992,** *68* (21), 3224-3227.

23. Datta, S., *Electronic transport in mesoscopic systems*. Cambridge university press: 1997.

24. Database(CSD), C. S., *version 2023. 2*. Cambridge Crystallographic Data Center: 2021.

25. Xiaoru, D.; Ben, Y.; Rui, Z.; Ruipu, W.; Yang, Z.; Yao, Z.; Zhenchao, D., Tip-induced bond weakening, tilting, and hopping of a single CO molecule on Cu(100). *Light: Advanced Manufacturing* **2022,** *3* (4), 729-738.

26. Okabayashi, N.; Peronio, A.; Paulsson, M.; Arai, T.; Giessibl, F. J., Vibrations of a molecule in an external force field. *Proceedings of the National Academy of Sciences* **2018,** *115* (18), 4571-4576.
